# Tolvaptan treatment improves survival of cirrhotic patients with ascites and hyponatremia

**DOI:** 10.1186/s12876-018-0857-0

**Published:** 2018-09-04

**Authors:** Shuzhen Wang, Xin Zhang, Tao Han, Wen Xie, Yonggang Li, Hong Ma, Roman Liebe, Honglei Weng, Hui-Guo Ding

**Affiliations:** 1 Department of Gastroenterology and Hepatology, Beijing You’an Hospital, Affiliated with Capital Medical University, Fengtai District, Beijing, 100069 China; 2 Department of Gastroenterology, Tianjin Third Central Hospital, Tianjin, China; 3 Department of Hepatology, Beijing Ditan Hospital, Affiliated with Capital Medical University, Beijing, China; 4 Department of Hepatology, PLA 302 Hospital, Beijing, China; 5 Liver Diseases Center, Beijing Friendship Hospital, Affiliated with Capital Medical University, Beijing, China; 6 Department of Medicine II, Section Molecular Hepatology, Medical Faculty Mannheim, Heidelberg University, Mannheim, Germany

**Keywords:** Ascites, Hyponatremia, Liver cirrhosis, Survival, Tolvaptan

## Abstract

**Background:**

Although tolvaptan treatment improves hyponatremia, only few studies have investigated whether tolvaptan actually benefits the survival of cirrhotic patients. This study evaluated the impact of tolvaptan on six-month survival of decompensated cirrhotic patients with and without hyponatremia.

**Methods:**

Two hundred forty-nine decompensated cirrhotic patients with or without hyponatremia were enrolled in a multicenter cohort study. Patients were divided into two groups according to receiving either tolvaptan or placebo treatment for 7-day. Subsequently, the patients were followed up for 6 months.

**Results:**

Two hundred thirty patients, including 98 with hyponatremia (tolvaptan vs. placebo: 69 vs. 29) finished the study. Tolvaptan did not alter serum sodium levels and survival outcome of decompensated cirrhotic patients without hyponatremia. However, tolvaptan treatment remarkably improved serum sodium levels and six-month survival in patients with hyponatremia. Following tolvaptan treatment, serum sodium levels were restored to normal in 63.8% of patients, whereas in patients receiving placebo, only 36.2% showed the same effect (*P* < 0.05). Compared to a six-month survival rate of 68.97% in patients receiving placebo, the survival rate in tolvapatan-treated patients was 89.94% (*P* < 0.05). Furthermore, six-month survival rate in the tolvaptan-treated hyponatremia patients with resolved serum sodium was 81.32%, whereas the survival in those with unresolved serum sodium was only 24% (*P* < 0.05).

**Conclusions:**

Tolvaptan improves short term survival in most decompensated cirrhotic hyponatremia patients with resolved serum sodium.

**Trials registration:**

Clinical trial one: ClinicalTrials.gov ID:NCT00664014, Registered on April 14, 2008.

Clinical trial two: ClinicalTrials.gov ID:NCT01349335, Registered on March 5, 2010.

Clinical trial three: ClinicalTrials.gov ID:NCT01349348, Registered on May 4, 2011.

## Background

Cirrhotic patients with ascites and hyponatremia have a poor quality of life and high mortality [1]. One-year survival rate in these patients is less than 60% [2]. Development of ascites with or without hyponatremia is associated with multiple pathophysiological alteration, i.e. renal water and sodium retention, hyperdynamic cardiovascular dysfunction secondary to arterial splanchnic vasodilation, activation of the renin-aldosterone system, and increased aldosterone and vasopressin levels in the peripheral circulation [3, 4].

Classic diuretics such as furosemide and aldosterone antagonists spironolactone improve water retention and edema, but exacerbate hyponatremia in cirrhotic patients [5]. Distinct from the classic diuretics, tolvaptan, a highly selective vasopressin V2 antagonist, effectively improves levels of serum sodium by increasing the excretion of electrolyte-free water without altering total level of electrolyte excretion [6]. Previous clinical studies showed that tolvaptan improved serum sodium levels in cirrhotic patients with ascites [6–8]. However, few studies to date have assessed whether tolvaptan treatment improves the survival of cirrhotic patients when hyponatremia is resolved [9]. The current cohort study addresses this question by following up cirrhotic patients for six months after tolvaptan treatment. The factors that influenced the efficacy of tolvaptan in liver cirrhotic patients with ascites are also analyzed.

## Methods

### Patients

**Fig. 1:**
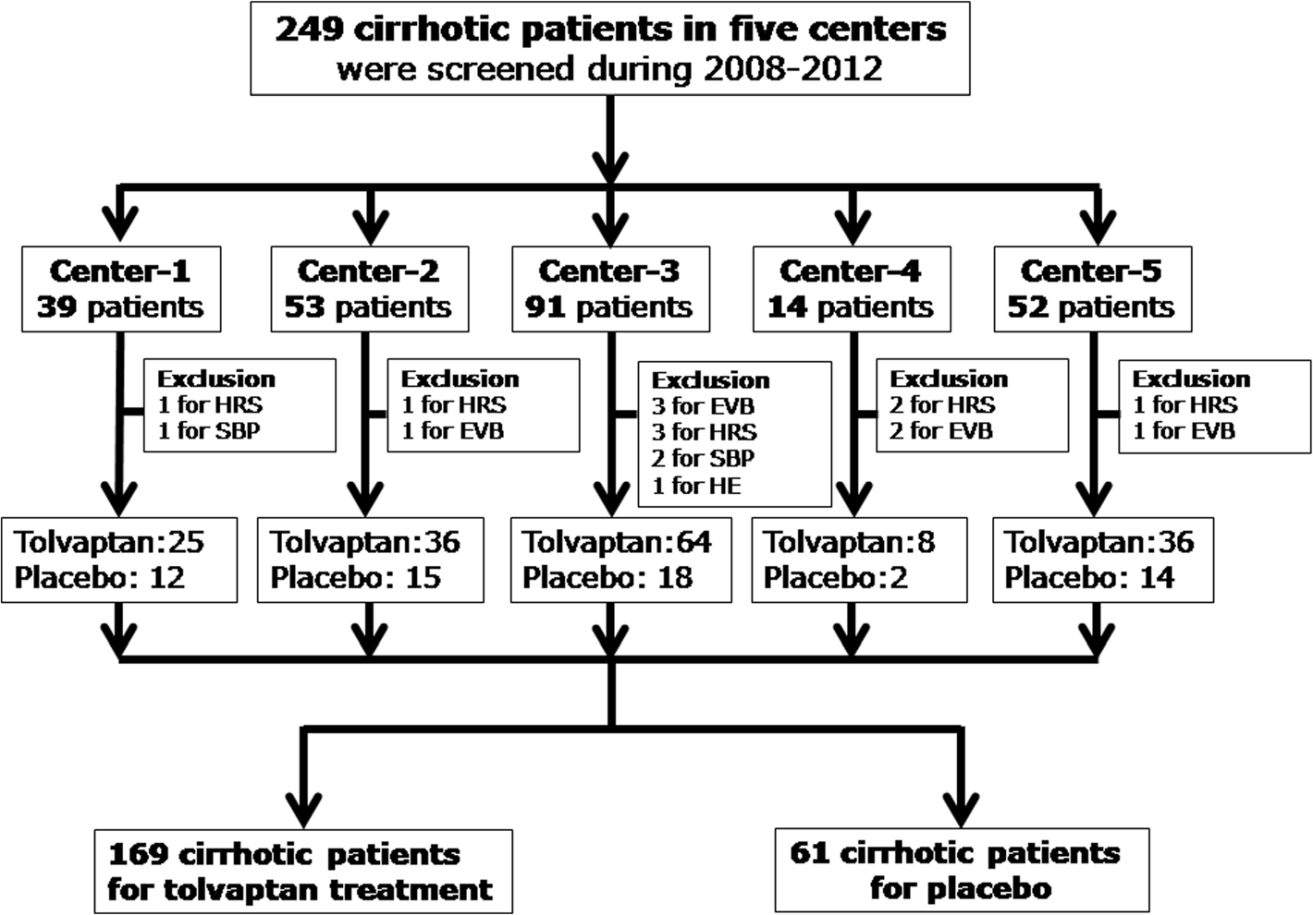
The flow chart of patients eligibility of the clinical trial

The prospective cohort enrolled 249 cirrhotic patients with ascites from three clinical trials, which were conducted in five medical centers between 2008 and 2012 (Fig. 1). The patients enrolled in three clinical trials were divided into two groups according to receiving tolvaptan (7.5 mg/day to 15 mg/day) or placebo for 7 days. The followings were a brief clinical trial descriptions. The first clinical trial performed from May 5, 2008 to July 7, 2009 was a multicenter, randomized (1:1), double-blind, placebo-controlled, which was designed to evaluate the efficacy and safety of the tolvaptan in patients with non-hypovolemic, and non-acute hyponatremia (ClinicalTrials.gov ID:NCT00664014). Cirrhotic patients with hyponatremia, defined as serum sodium< 135 mmol/L, were hospitalized to receive drug administration. They received 15 mg/day tolvaptan or placebo for the first 4 days. After 4 days, the dose was increased to 30 mg/day and 60 mg/day in the patients whose serum sodium levels did not respond. The remaining patients remained on the same dosage as on days 1–4 until the 7th day.. The second clinical trial, which was performed from 2009 to 2010, was a multicenter, open-label, double-blinded, and placebo-controlled design (ClinicalTrials.gov ID:NCT01349335) [10]. The aim of the trial was to evaluate the effect of tolvaptan in cirrhotic patients with ascites. The patients enrolled in this trial had insufficient response to combination diuretics treatment (furosemide ≥40 mg/day and spironolactone ≥20 mg/day; or furosemide ≥20 mg/day and spironolactone ≥40 mg/day). Patients were randomly assigned to three groups (1:1:1) receiving placebo, tolvaptan 15 mg/day and tolvaptan 30 mg/day for 7 days, respectively. The third clinical trial, which was performed between October 5, 2010 and January 20, 2012, was a multicenter randomized, double-blind, and placebo-controlled study (ClinicalTrials.gov ID:NCT01349348). This trial evaluated the efficacy and safety of tolvaptan in cirrhotic patients with ascites. Patients who had insufficient response to combination therapies of diuretics were treated with tolvaptan 7.5 mg/day, tolvaptan 15 mg/day or placebo (1:2:1) for 7 days, respectively. Patients were followed up for 6 months after treatment. The endpoints of the study were (1) survival of patients and (2) resolved serum sodium levels. Normal serum sodium was defined as serum sodium concentration between 135 and 155 mmol/L. Mild, moderate and severe hyponatremia were considered when levels of serum sodium between 130 and 135 mmol/L, 125 and 130 mmol/L, and less than 125 mmol/L, respectively (Table 1). “Resolved serum sodium” was defined as a patient with hyponatremia before treatment restoring levels of serum sodium to between 135 and 155 mmol/L after treatment.

**Table 1:** The baseline characteristics of the patients enrolled in this study

Index	Tolvaptan (*n* = 169)	Placebo (*n* = 61)	*P* value
Age (mean ± SD)	54.6 ± 8.58	51.6 ± 10.71	0.03
Gender (F/M)	33/136	12/49	0.98
Etiology of cirrhosis (n,%)			
Hepatitis B	102 (60.3)	41 (67.3)	0.03
Hepatitis C	9 (5.3)	3 (4.9)	
Alcohol liver disease	33 (19.5)	11 (18.0)	
PBC	2 (1.2)	0 (0)	
Mixed^a^	13 (7.7)	5 (8.2)	
Cryptogenic	4 (2.4)	0 (0)	
Others^b^	6 (3.6)	1 (1.6)	
Child-Pugh grade (n, %)			
A (5–6)	2 (1.2)	1 (1.6)	0.36
B (7–9)	113 (66.8)	45 (73.8)	
C (10–15)	54 (32.0)	15 (24.6)	
Edema of lower limbs (n, %)			
No	123 (72.8)	43 (70.5)	0.86
Yes	46 (27.2)	18 (29.5)	
Serum sodium (mmol/L)			0.56
Median	135.8	135.0	
(Min, Max)	(117.2, 146.3)	(124.8, 142.7)	
Normal serum sodium (n, %) (≥ 135 and < 150 mmol/L)	100 (59.2)	32 (52.5)	
Mild hyponatremia (n, %) (≥ 130 and < 135 mmol/L)	44 (26.0)	21 (34.4)	
Middle hyponatremia (n, %) (≥ 125 and < 130 mmol/L)	19 (11.2)	7 (11.5)	
Severe hyponatremia (n, %) (< 125 mmol/L)	6 (3.6)	1 (1.6)	
Serum potassium (mmol/L)	4.2 ± 0.5	4.1 ± 0.7	0.98
Serum urea nitrogen (mmol/L)	5.3 ± 0.8	5.6 ± 0.8	0.93

^a^Mixed etiology: Hepatitis B + alcoholic hepatitis, hepatitis B + primary biliary cirrhosis (PBC), hepatitis C + alcoholic hepatitis, alcoholic hepatitis+ PBC. PBC:primary biliary cirrhosis

^b^Other etiology: 3 drug-induced hepatitis, 2 cardiac disease, 2 autoimmune liver disease

Decompensated cirrhotic patients were enrolled according to the following criteria: (1) aged between 18 and 75 years, (2) definite history of cirrhosis, (3) signs and symptoms of decompensated cirrhosis, such as splenomegaly, hypersplenism, variceal bleeding, ascites, and hepatic encephalopathy, (4) confirmed cirrhosis by image measurements, i.e. B-ultrasound scanning (LOGIQ9; GE Company, Fairfield, United States) and computerized tomography (CT; GE HISPEED DXI; GE Company) and (5) consenting to follow-up for six months.

Patients with the following diseases were excluded: (1) type I hepatorenal syndrome, (2) nervous system diseases such as Alzheimer’s disease, Parkinson’s disease, multiple sclerosis, epilepsy, Guillain-Barré syndrome, etc., (3) poorly controlled diabetes (fasting glucose > 220 mg/dL), (4) heart failure with ascites, (5) anuria (urine volume < 100 mL/day), (6) dysuresia, (7) hepatic encephalopathy ≥ Grade II, (8) hepatocellular carcinoma, (9) chronic liver failure, (10) cerebrovascular accident within 30 days prior to the study medication, and (11) history of cerebral infarction or stroke.

The study protocol was performed in compliance with the Declaration of Helsinki and approved by the Ethics Committees of Beijing Youan Hospital affiliated to Capital Medical University. Signed informed consent was obtained from each patient for using samples, materials and publication.

### Clinical and laboratory data

Symptoms, signs, and adverse events were recorded. Serum markers for hepatitis B and C viruses were detected by electrochemiluminescence immunoassay (Roche E170 modular immunoassay analyzer, Roche Diagnostics, Mannheim, Germany). Serum sodium and potassium levels, liver and renal function, including serum alanine aminotransferase (ALT), aspartate aminotransferase (AST), total bilirubin (TBIL), albumin, creatinine, and urea nitrogen, were measured with an automatic biochemical analyzer (AU5400, Olympus Company, Tokyo, Japan).

### Safety

Patients were routinely monitored throughout the study. Any occurrences of adverse events and deaths were recorded.

### Statistical analysis

Student’s t test, χ2, and Fisher’s exact test were used to test the intergroup difference according to the types of variables. The Kaplan–Meier estimator and log-rank test were used to calculate the survival rate. A *P* value less than 0.05 (two-way) was considered statistically significant.

## Results

### Study patients

After 6-month of follow-up, 230 patients finished the study. Among them, 169 patients were treated with tolvaptan and 61 with placebo. The baseline characteristics of the enrolled patients were shown in Table 1. Except for age and etiology, there were no differences of demographic and clinical parameters, i.e. gender, Child-Pugh grades, edema, levels of serum sodium, potassium and urea nitrogen, between the two groups of patients at the baseline of the clinical study. The mean age of patients treated with tolvaptan and with placebo were 54 and 51 years, respectively (*P* < 0.05). The major etiology in both groups was chronic hepatitis B infection (60.3% vs. 67.3%, *P* < 0.05).

#### Tolvaptan does not impact serum sodium levels of cirrhotic patients without hyponatremia

First we investigated whether tolvaptan impacted serum sodium levels in cirrhotic patients without hyponatremia. This clinical study enrolled 132 cirrhotic patients with normal serum sodium levels. Among these, 100 patients received tolvaptan treatment and 32 placebo (Table 1). The dynamic alteration of serum sodium levels in cirrhotic patients treated with tolvaptan or placebo were shown in Table 2. Tolvaptan treatment for 7 days increased mean values of serum sodium from 138.15 ± 2.58 mmol/L to 139.83 ± 3.32 mmol/L (*P* < 0.05). In the period of follow-up levels of serum sodium in these patients decreased compared to those at the baseline (*P* < 0.05), however, the concentrations of serum sodium during the follow-up still remained within the normal range: 136.88 ± 5.09 mmol/L after 3 months and 136.97 ± 6.92 mmol/L after 6 months. Placebo treatment for 7 days did not alter serum sodium levels of cirrhotic patients: 138.60 ± 2.24 mmol/L vs. 138.23 ± 2.16 mmol/L (*P* > 0.05). During the follow-up, levels of serum sodium in these patients reduced to 136.97 ± 4.18 mmol/L at 3 months and 133.18 ± 9.31 mmol/L (*P* > 0.05).

**Table 2:** Dynamic alteration of serum sodium levels in cirrhotic patients treated with tolvaptan or placebo

	Tolvaptan (*n* = 169)					Placebo (*n* = 61)				
	0 day	4 days	7 days	3 months	6 months	0 day	4 days	7 days	3 months	6 months
Normal sodium, mmol/L (n)	138.15 ± 2.58 (100)	140.28 ± 3.07 (95)	139.83 ± 3.32* (95)	136.88 ± 5.09* (56)	136.97 ± 6.92* (49)	138.60 ± 2.24 (32)	138.52 ± 2.39 (29)	138.23 ± 2.16 (24)	136.97 ± 4.18 (14)	133.18 ± 9.31 (11)
Mild hyponatremia, mmol/L (n)	132.98 ± 1.31 (44)	138.02 ± 3.11** (44)	136.59 ± 3.51** (41)	133.78 ± 4.13** (33)	131.96 ± 5.29** (23)	131.96 ± 1.44 (21)	131.34 ± 3.03 (13)	131.06 ± 4.65 (13)	129.45 ± 6.66 (11)	134.50 ± 6.20 (9)
Middle hyponatremis, mmol/L (n)	127.35 ± 1.17 (19)	131.50 ± 2.85** (18)	132.29 ± 2.87** (18)	132.24 ± 5.71** (11)	129.81 ± 7.02** (10)	127.94 ± 0.92 (7)	130.30 ± 3.82 (5)	129.20 ± 10.32 (3)	134.30 (2)	139.30 (2)
Severe hyponatremia, mmol/L (n)	120.87 ± 3.33 (6)	126.87 ± 7.05** (6)	126.50 ± 6.61** (5)	131.10 ± 6.15** (3)	132.30 (2)	124.80 (1)	ND	ND	ND	ND

Compared with 0 day, *P > 0.05, **P < 0.01

#### Tolvaptan improves serum sodium levels of cirrhotic patients with hyponatremia

Next, we assessed the effect of tolvaptan on cirrhotic patients with hyponatremia. In this study, 69 cirrhotic patients (44 with mild, 19 with moderate and 16 with severe hyponatremia) received tolvaptan treatment while 29 (21 with mild, 7 with moderate and 1 with severe hyponatremia) were treated with placebo.

Tolvaptan treatment for 7 days significantly improved levels of serum sodium of cirrhotic patients with hyponatremia (Table 2). Thirty-two patients (72.7%) with mild, 10 (52.6%) with moderate and 2 (33.3%) with severe hyponatremia restored serum sodium levels to normal levels. The mean value of serum sodium in mild, moderate and severe hyponatremia patients with tolvaptan treatment for 7 days increased from 132.98 ± 1.31, 127.35 ± 1.17 and 120.87 ± 3.33 mmol/L to 136.59 ± 3.51, 132.29 ± 2.87 and 126.50 ± 6.61 mmol/L, respectively (*P* < 0.01 for all). Three months after tolvaptan treatment, levels of serum sodium in patients with mild, moderate and severe hyponatremia remained at 133.78 ± 4.13, 132.24 ± 5.71 and 131.10 ± 6.15 mmol/L, respectively (*P* < 0.01). At the end of follow-up, levels of serum sodium in 23 patients with mild hyponatremia had decreased to 131.96 ± 5.29 mmol/L (*P* < 0.01,). However, 10 patients with moderate and 2 with severe hyponatremia kept at 129.81 ± 7.02 and 132.30 mmol/L, higher than the baseline levels (*P* < 0.01).

Placebo treatment did not alter levels of serum sodium in patients with hyponatremia (Table 2). One patient with severe hyponatremia died 2 days after hospitalization. The mean values of serum sodium in patients with mild and moderate hyponatremia before and after placebo treatment were 131.96 ± 1.44 vs. 131.06 ± 4.65 mmol/L and 127.94 ± 0.92 vs. 129.20 ± 10.32 mmol/L, respectively (*P* > 0.05). Only 9 patients with mild and 2 patients with moderate hyponatremia in this group finished follow-up. The mean value of serum sodium in patients with mild hyponatremia was 134.50 ± 6.20 mmol/L (*P* > 0.05 compared to the baseline).

#### Tolvaptan improves six-month survival of cirrhotic patients with hyponatremia

**Fig. 2:**
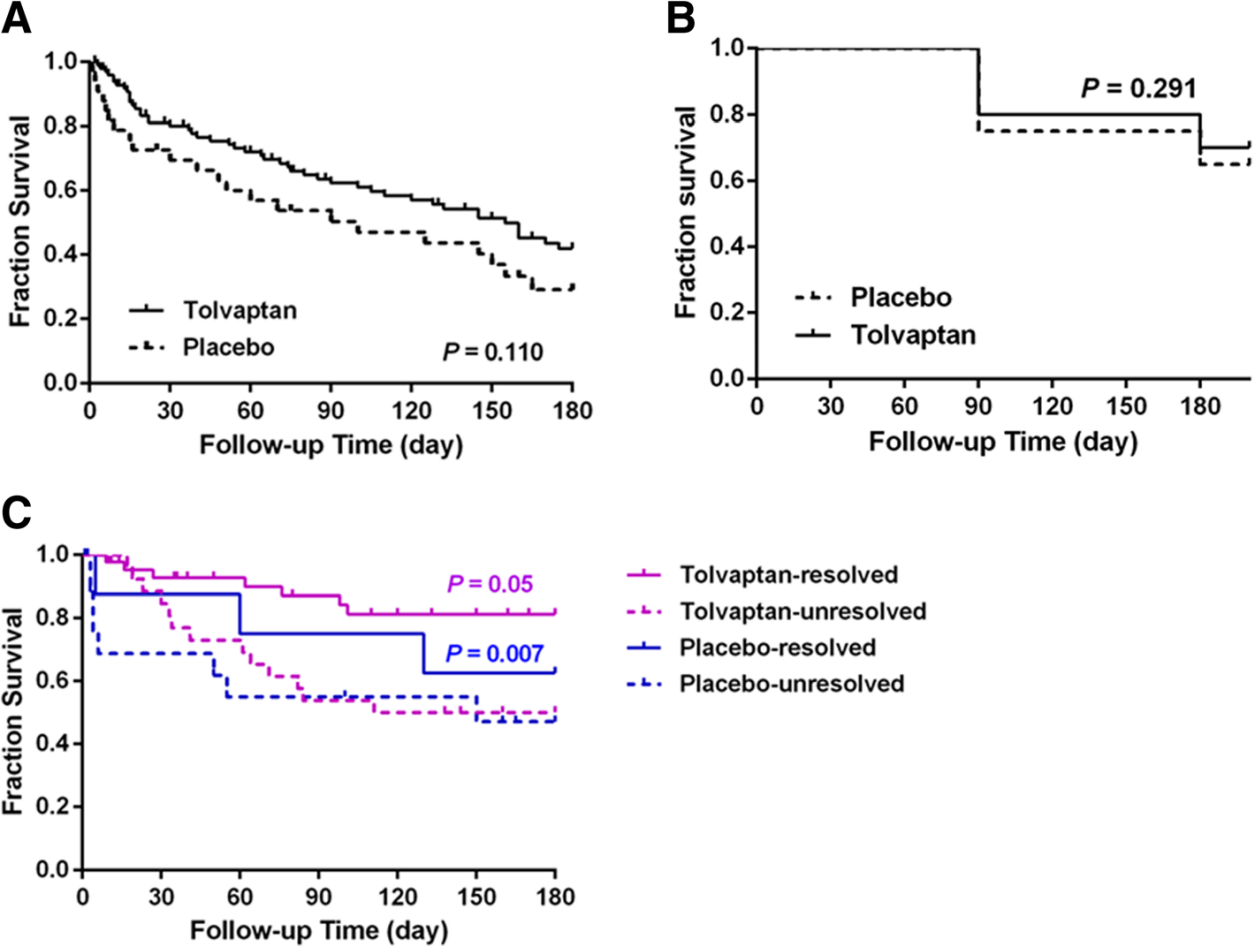
Six-month survival of cirrhotic patients with ascites who received tolvaptan or placebo treatment. The Kaplan–Meier survival curves was not statistically significant difference in patients without hyponatremia between tolvaptan and placebo treatment (*P* > 0.05, **a**), also in cirrhotic patients with hyponatremia(*P* > 0.05, **b**).However, the survival of patients with resolved serum sodium was significantly higher than of those unresolved serum sodium either tolvaptan or placebo treatment(*P* < 0.01, **c**)

Next, we examined the six-month survival of cirrhotic patients. First we analyzed the impact of tolvaptan treatment on survival of cirrhotic patients without hyponatremia. Although tolvaptan-treated patients showed higher six-month survival rate than placebo-treated ones, the difference was not statistically significant (*P* > 0.05, Fig. 2a). The differences in three or six-month survival rates of cirrhotic patients with hyponatremia between tolvaptan treated patients and control patients were not statistically significant either (P > 0.05, Fig. 2b). Compared to patients receiving placebo, however, three or six-month survival rates in the hyponatremia patients receiving tolvaptan increased from 75.87% (22/29) and 68.97% (19/29) to 91.31% (63/69) and 89.94%, respectively.

In hyponatremia patients receiving tolvaptan, survival depended on the serum sodium responded to tolvaptan treament or not. The three or six-month survival rates in hyponatremia patients with restored normal serum sodium (tolvaptan responders) reached 86.36% (38/44) and 81.32% (36/44), whereas in those with unrestored serum sodium levels (non-responders) were a mere 40% (10/25) and 24% (6/25) (*P* < 0.05).

Notably, even in the hyponatremia patients receiving placebo, survival of the patients depended on restoring serum sodium levels to normal during follow-up. The six-month survival rate of patients with restored serum sodium levels (62.5%) was significantly higher than of those without (28.7%), *P* < 0.01. The Kaplan–Meier survival curves shown that the survival of patients with resolved serum sodium was significantly higher than of those unresolved serum sodium either tolvaptan or placebo treatment (Fig. 2c).

#### Safety assessment

No side-effects were detected during treatment and follow-up of this study. Monitoring liver function did not detect any tolvaptan-associated alterations in serum ALT and TBIL levels (data not shown).

## Discussions

Hyponatremia is tightly associated with cirrhotic complications, including hepatic encephalopathy, refractory ascites, renal failure, spontaneous bacterial peritonitis, and hepatic hydrothorax, and concomitant high mortality [11]. Tolvaptan is a recently FDA approved drug used to treat hyponatremia in cirrhotic patients. Given only several clinical trials of tolvaptan have been published to date [11–16], including for treatment of liver cirrhotic patients with refractory ascites [17], the safety and efficacy of this drug in cirrhotic patients are not clarified yet. It is unknown to date whether tolvaptan treatment improves the survival of cirrhotic patients. It is also not clear whether tolvaptan has similar or different efficacy in cirrhotic patients with different degree of hyponatremia. A single-center retrospective study in Japan included 95 cirrhotic patients who received tolvaptan for ascites treatment [9]. Among patients with hyponatremia (serum sodium level < 135 mEq/L), 60.0% achieved a normal level after 1 week treatment, and the survival rate was significantly higher in patients with a normalized serum sodium level.

In this prospective cohort study, which comprised 249 cirrhotic patients with ascites from three clinical trials and five medical centers, we investigated the impact of tolvaptan on six-month mortality of cirrhotic patients with different levels of serum sodium. We found that low dosage of tolvaptan (7.5 mg/day or 15 mg/day) treatment for 7 days significantly improved six-month survival of decompensated cirrhotic patient with hyponatremia. Compared to placebo-treated patients whose six-month survival rate was 68.97%, 89.94% of decompensated cirrhotic patients with hyponatremia survived after tolvaptan treatment. Survival in tolvaptan-treated patients was determined by whether they responded to tolvapatan or not. Hyponatremic patients who restored normal levels of serum sodium after tolvaptan treatment (tolvaptan responders) demonstrated a six-month survival rate of 89.31%, whereas those with refractory hyponatremia (non-responders) only had a 24% survival rate. The baseline serum sodium levels were closely associated with the efficacy of tolvaptan. Compared to the impressive benefit of tolvaptan on cirrhotic patients with mild and moderate hyponatremia whose six-month survival rate was more than 50%, the drug seems to have limited effects on cirrhotic patients with severe hyponatremia. Among 6 patients with severe hyponatremia, only 2 patients survived and maintained normal serum sodium levels 6 months after tolvaptan treatment. The result is consistent with two previous studies [18, 19]. It is unknown why severe hyponatremia in cirrhotic patients is difficult to treat with tolvaptan. The existence of an adaptive renal response to chronic hyponatremia, which results in a diminished response to a selective antagonist such as tolvaptan, might be an explanation [20].

In contrast to its efficacy of tolvaptan in cirrhotic patients with hyponatremia, tolvaptan did not significantly alter serum sodium concentration and improve survival in cirrhotic patients without hyponatremia. Interestingly, placebo-treated patients who recovered from hyponatremia had significantly higher survival outcome than those who didn’t. These results argue a crucial role of restoring hyponatremia for cirrhosis recovery. More than 80% hyponatremic patients who did not respond to tolvaptan treatment died of hepatorenal syndrome (data not shown). Therefore, it is very important to predicte the therapeutic response to tolvaptan in clinical practice. Several recent clinical studies showed that serum BUN/Cr ratio ≥ 17.5, urine Na/K ratio < 3.09, and decreased urinary aquaporin 2 levels were predictive of being non-responsiveness to tolvaptan [21–23]. It is not totally clear why a short tolvaptan administration can improve survival in cirrhotic patients. Based on current knowledge, hyponatremia is the most important factors affecting the prognosis of patients with cirrhosis and ascites [2, 11]. Resolved hyponatremia may directly reduce the risk of hepatic encephalopathy, hepatorenal syndrome and spontaneous peritonitis in decompensated cirrhotic patients, which may directly improve survival in patients who respond to tolvaptan treatment [9]. This might partially explain why the survival is significantly improved in resolved hyponatremia patients following tolvaptan treatment.

Considering the safety of patients, this clinical trial adopted low dosages of tolvaptan. These dosages are safe. No tolvaptan-associated side effects were reported in this study. Notably, whether increasing dosage can improve the efficacy of tolvaptan and thus increase response rate or survival rates in responders should be further investigated in the future clinical studies.

## Conclusions

Taken together, we found that the six-month survival may be improved in cirrhotic patients with hyponatremia after tolvaptan short administration, particularly in tolvaptan responders. Severity of hyponatremia in cirrhotic patients impacts the response of tovalptan. The underlying mechanisms should be further investigated.

## Abbreviations

ALT: Alanine aminotransferase
AST: Aspartate aminotransferase
TBIL: Total bilirubin

## References

[1] Moore CM, Van Thiel DH (2013). Cirrhotic ascites review: pathophysiology, diagnosis and management. World J Hepatol.

[2] Yu C, SharmaN SS (2013). Hyponatremia: clinical associations, prognosis, and treatment in cirrhosis. ExpClin Transplant.

[3] Gordon FD (2012). Ascites. Clin Liver Dis.

[4] Runyon BA, AASLD (2013). Introduction to the revised American Association for the Study of Liver Diseases Practice Guideline management of adult patients with ascites due to cirrhosis, 2012. Hepatology.

[5] Leiva JG, Salgado JM, Estradas J, Torre A, Uribe M (2007). Pathophysiology of ascites and dilutional hyponatremia: contemporary use of aquaretic agents. Ann Hepatol.

[6] Josiassen RC, Curtis J, Filmyer DM, Audino B, Skuban N, Shaughnessy RA (2010). Tolvaptan: a new tool for the effective treatment of hyponatremia in psychotic disorders. Expert Opin Pharmacother.

[7] Sakaida I, Kawazoe S, Kajimura K, Saito T, Okuse C, Takaguchi K (2014). Tolvaptan for improvement of hepatic edema: a phase 3, multicenter, randomized, double-blind, placebo-controlled trial. Hepatol Res.

[8] Berl T, Quittnat-Pelletier F, Verbalis JG, Schrier RW, Bichet DG, Ouyang J (2010). Oral tolvaptan is safe and effective in chronic hyponatremia. J Am SocNephrol.

[9] Kogiso T, Kobayashi M, Yamamoto K, Ikarashi Y, Kodama K, Taniai M (2017). The outcome of cirrhotic patients with ascites is improved by the normalization of the serum sodium level by tolvaptan. Intern Med.

[10] Wang YF, Tang JT, Han T, Ding HG, Ye WJ, Wang MR (2018). Tolvaptan in Chinese cirrhotic patients with ascites: a randomized, placebo-controlled phase 2 trial. J Dig Dis.

[11] Umemura T, Shibata S, Sekiguchi T, Kitabatake H, Nozawa Y, Okuhara S (2015). Serum sodium concentration is associated with increased risk of mortality in patients with compensated liver cirrhosis. Hepatol Res.

[12] Cárdenas A, Ginès P, Marotta P (2012). The safety and efficacy of tolvaptan, an oral vasopressin antagonist in the treatment of hyponatremia in cirrhosis. J Hepatol.

[13] Yan L, Xie F, Lu J, Ni Q, Shi C, Tang C, Yang J (2015). The treatment of vasopressin V2-receptor antagonists in cirrhosis patients with ascites: a meta-analysis of randomized controlled trials. BMC Gastroenterol.

[14] Kogiso T, Tokushige K, Hashimoto E, Ikarashi Y, Kodama K, Taniai M (2016). Safety and efficacy of long-term tolvaptan therapy for decompensated liver cirrhosis. Hepatol Res.

[15] Zhang X, Wang SZ, Zheng JF, Zhao WM, Li P, Fan CL (2014). Clinical efficacy of tolvaptan for treatment of refractory ascites in liver cirrhosis patients. World J Gastroenterol.

[16] Akiyama S, Ikeda K, Sezaki H, Fukushima T, Sorin Y, Kawamura Y (2015). Therapeutic effects of short- and intermediate-term tolvaptan administration for refractory ascites in patients with advanced liver cirrhosis. Hepatol Res.

[17] Wang SZ, Ding HG (2017). New therapeutic paradigm and concepts for patients with cirrhotic refractory ascites. Zhonghua Gan Zang Bing Za Zhi.

[18] Pose E, Solà E, Piano S, Gola E, Graupera I, Guevara M (2017). Limited efficacy of Tolvaptan in patients with cirrhosis and severe Hyponatremia: real-life experience. Am J Med.

[19] Ahluwalia V, Heuman DM, Feldman G, Wade JB, Thacker LR, Gavis E (2015). Correction of hyponatraemia improves cognition, quality of life, and brain oedema in cirrhosis. J Hepatol.

[20] Esteva-Font C, Baccaro ME, Fernández-Llama P, Sans L, Guevara M, Ars E (2006). Aquaporin-1 and aquaporin-2 urinary excretion in cirrhosis: relationship with ascites and hepatorenal syndrome. Hepatology.

[21] Nakai M, Ogawa K, Takeda R, Ohara M, Kawagishi N, Izumi T (2018). Increased serum C-reactive protein and decreased urinary aquaporin 2 levels are predictive of the efficacy of tolvaptan in patients with liver cirrhosis. Hepatol Res.

[22] Komiyama Y, Kurosaki M, Nakanishi H, Takahashi Y, Itakura J, Yasui Y (2017). Prediction of diuretic response to tolvaptan by a simple, readily available spot urine Na/K ratio. PLoS One.

[23] Kawaratani H, Fukui H, Moriya K, Noguchi R, Namisaki T, Uejima M (2017). Predictive parameter of tolvaptan effectiveness in cirrhotic ascites. Hepatol Res.

